# Adaptive Parameters for LoRa-Based Networks Physical-Layer

**DOI:** 10.3390/s23104597

**Published:** 2023-05-09

**Authors:** Edelberto Franco Silva, Lucas M. Figueiredo, Leonardo A. de Oliveira, Luciano J. Chaves, André L. de Oliveira, Denis Rosário, Eduardo Cerqueira

**Affiliations:** 1 Department of Computer Science, Federal University of Juiz de Fora (UFJF), Juiz de Fora 36036-330, MG, Brazilandre.oliveira@ice.ufjf.br (A.L.d.O.); 2 Graduate Program in Computer Science—UFJF, Juiz de Fora 36036-330, MG, Brazil; 3 Faculty of Computer Engineering (ENGCOMP), Federal University of Pará (UFPA), Belém 66075-110, PA, Brazil

**Keywords:** LoRa, IoT, Sub-GHz, wireless network, wireless cognitive radio

## Abstract

Sub-GHz communication provides long-range coverage with low power consumption and reduced deployment cost. LoRa (Long-Range) has emerged, among existing LPWAN (Low Power Wide Area Networks) technologies, as a promising physical layer alternative to provide ubiquitous connectivity to outdoor IoT devices. LoRa modulation technology supports adapting transmissions based on parameters such as carrier frequency, channel bandwidth, spreading factor, and code rate. In this paper, we propose SlidingChange, a novel cognitive mechanism to support the dynamic analysis and adjustment of LoRa network performance parameters. The proposed mechanism uses a sliding window to smooth out short-term variations and reduce unnecessary network re-configurations. To validate our proposal, we conducted an experimental study to evaluate the performance concerning the Signal-to-Noise Ratio (SNR) parameter of our SlidingChange against InstantChange, an intuitive mechanism that considers immediate performance measurements (parameters) for re-configuring the network. The SlidingChange is compared with LR-ADR too, a state-of-the-art-related technique based on simple linear regression. The experimental results obtained from a testbed scenario demonstrated that the InstanChange mechanism improved the SNR by 4.6%. When using the SlidingChange mechanism, the SNR was around 37%, while the network reconfiguration rate was reduced by approximately 16%.

## 1. Introduction

Society has been changing dramatically recently through disruptive Internet of Things (IoT) technology [1]. Currently, IoT is receiving a lot of attention from both academia and industry due to its enormous potential to create a wide range of innovative applications [2] for Smart Cities [1,3,4,5], Smart Farms [6,7,8,9], and Long-Range Machine-to-Machine (LRM2M) [10]. Those applications rely on wireless communication technology to collect and transmit data to a central server for further analysis. Therefore, communication is an important issue for the massive adoption and deployment of IoT applications [11].

With recent advances in wireless networks, long-range communication technologies such as Low-Power Wide-Area Networks (LPWAN) and Sub-GHz have drawn the attention of researchers and companies around the world. Such technologies serve as a subsidy for IoT applications by providing high coverage with low energy consumption [12]. In this context, the Long Range (LoRa^®^) technology has gained considerable prominence, due to its robustness and low energy consumption, without sacrificing its signal range and coverage. LoRa is a proprietary radio-frequency modulation technology from Semtech (http://www.semtech.com, accessed on 10 December 2022) company, which relies on Chirp Spread Spectrum (CSS) modulation [12]. On the other hand, LoRa Wide Area Networks (LoRaWAN) consider LoRa radio as the physical layer, and it defines the upper layers, and the network architecture [13]. Chirp modulation can be illustrated by a pulse, which is a concept used by animals, such as dolphins, or radars, and wireless for communication. CSS technology supports the Shannon–Hartley theorem to enable more robust communication against interference.

As communication technology adoption in a given frequency spectrum increases, the transmission medium dispute and the interference intensity also increase. This issue reduces the packet delivery probability, and thus there is a need for using the frequency spectrum to increase the network efficiency as a whole [14]. In the case of LoRa technology, its three operating frequency bands are discontinuous, making the adoption of an aggregation channel or channel shift strategies complicated compared to IEEE 802.11 networks. Furthermore, inter-symbolic interference between channels within one of these frequency bands can be equally harmful to the network as a whole, due to LoRa considering a spread-spectrum modulation scheme.

The concept of Cognitive Radio (CR), supported by the concurrent advances in the development of Software Defined Radio (SDR) technologies [15], allows a radio or a system to sense its operational electromagnetic environment and dynamically adjust its operating parameters to maximize throughput, mitigate interference, supporting interoperability, and accessing secondary markets [16]. As a consequence of the potential use of free spectra for opportunistic communications, contributing to the massive adoption of IoT applications, research and development on CR technologies have grown exponentially recently. Cognitive Radio-based technologies support *adaptive and autonomous spectral awareness* (spectrum sensing (SS)), *detection* of available channels (spectrum decision making), *dynamic adjustment* of radio operating parameters (spectrum mobility), and conduct *concurrent communication* (spectrum sharing).

In this context, cognitive networks and adaptive exchanges of network operating ranges become an interesting alternative for improving physical parameters of network quality over time [14,17,18]. This raises the following research question (**RQ1**): How can cognitive radio and adaptive exchange of networks be combined to reduce inter-symbolic interference between channels in LoRa networks? In order to answer this question, we propose and evaluate a novel technique, named *SlidingChange*, to support automated decision-making on frequency shifts for maximizing SNR, decreasing the Bit Error Rate (BER), and the number of frequency changes.

In earlier work [14], the authors presented the *InstantChange* method to improve SNR, BER, and frequency change parameters. However, the *InstantChange* method only considers the values of the most recent SNR samples of LoRa messages in 433 MHz and 915 MHz frequencies for decision making. In this article, we extend our previous work with the proposal of a novel *SlidingChange* algorithm considering an average of $W_{n}-1$ ($W_{n}$ representing the size of the sliding window to be used), previous measurements, and the current measurement to dampen punctual effects and impulsive noise that can happen in the transmission environment. We also evaluate the effectiveness of the *SlidingChange* algorithm in LoRa networks to optimize the SNR considering the shifts and overhead in the decision-making process, opening an interesting research area complementary to Cognitive Radios in LoRa and LoRaWAN Physical-Layer optics.

The main contributions of this article can be summarized as follows:

- Verify the possibility of changing the configuration ’parameters’ in LoRa on the fly to increase the network performance in terms of SNR and BER;
- Propose and validate a novel algorithm with adaptive parameters considering the history of configuration shifts, creating a smarter cognitive-radio for LoRa;
- Compare the state of the art with the proposal using a real dataset.

The remainder of this article is organized as follows: Section 2 presents the related work. Section 3 describes the proposed SlidingChange mechanism to improve the quality of LoRa communication in a theoretical format. Section 4 illustrates the results of practical experiments based on the theory presented for the proposal of sliding windows. Finally, Section 5 presents the conclusion and future research directions.

## 2. Related Work

Related research on this topic comprises: **(i)** empirical studies that evaluate performance and other quality attributes of resource allocation strategies (Bor et al. [19]), **(ii)** proposals of research allocation strategies (Bianchi et al. [5], Goldoni et al. [20], Lima et al. [11], Reynders et al. [21], and Ramli et al. [8]), approaches (Valach and Macko [22]), methods (Moraes et al. [23], and Slabicki et al. [24]), techniques (Jeon and Jeong [25], and Figueiredo and Franco [14]), and algorithms (Abdelfadeel et al. [17], and Farhad et al. [26]), and **(iii)** studies reporting both proposals of resource allocation strategies and empirical evaluation results (Bianchi et al. [5], Valach and Macko [22], Moraes et al. [23], Aneda et al. [27], and Moysiadis et al. [28]). The contributions of each one of these studies and a comparative analysis are detailed in the following.

Bor et al. [19] conducted an experimental evaluation on how Spread Factor (SF) configuration impacts the network performance in an urban area. They found that the network’s scalability increases as soon as the SF is set to provide a lower message’s air time, which has less frequency spectrum usage. SF is helpful in increasing scalability and could be used to change the LoRa’s parameters dynamically. However, despite the promising results, the study did not demonstrate the effectiveness of the proposed technique in a realist environment (i.e., only running the proposal in a simulated environment).

Bianchi et al. [5] proposed EXPLoRa-C, a novel resource allocation strategy that considers the spreading factor balancing, the channel capture effect, and network topology to make resource allocation decisions. The authors evaluated the performance of EXPLoRa-C and existing resource allocation mechanisms on LoRa networks considering both perfect and imperfect channel orthogonality for different SF values. In their study, the authors conducted the performance analysis via simulations in both single-gateway and multi-gateway scenarios using data from a realist LoRaWAN network with 268 water meters. The results demonstrate that the EXPLoRa-C resource allocation strategy improves up to 38% of network capacity compared with the Adaptive Data Rate (ADR) allocation strategy, and it seems to be more robust to different operating/load conditions and network topology configurations.

Valach and Macko [22] developed an approach to LoRa parameter adaption based on machine learning techniques to improve communication in IoT networks. The authors adapted the existing LoRa@FIIT algorithm using machine learning-based adaptiveness to network condition changes to achieve energy-efficient communication. The authors conducted a simulation-based experiment to evaluate the proposed implementation of the LoRaFIIT algorithm in a network with end nodes and a gateway connected to a real network server. The results demonstrated significant reduction in the number of collisions for mobile nodes, reducing the channel congestion, and improving energy-efficiency by avoiding re-transmissions.

Goldoni et al. [20] introduced a resource allocation strategy that correlates the RSSI values with the weather conditions. The authors deployed a LoRaWAN network containing one (1) LoRa gateway and eight (8) LoRa end devices on a vineyard area in Italy. The data were collected for approximately 85 days. The end devices generated network traffic, and their RSSI values, measured by the gateway, were recorded together with the data of a weather station.

Lima et al. [11] focus on Quality of Service (QoS) and energy efficiency of IoT applications in dense scenarios. They propose the ARPA adaptive priority-aware resource allocation mechanism to support real-time adjustment of radio-related parameters to improve scalability and ensure QoS of IoT devices to achieve energy efficiency. The authors evaluated the ARPA resource allocation mechanism via simulation. The simulation results demonstrated a 95% of improvement in the energy efficiency of IoT devices, with higher rates of packet delivery and reduced delay in high-priority applications. Moraes et al. [23] proposed an adaptive resource allocation based on mixed-integer linear programming to define the best LoRanWAN parameter settings to reduce channel utilization and maximize the number of packets delivered. The authors evaluated their solution against resource allocation heuristics. The results demonstrated the effectiveness of resource allocation heuristics, achieving results nearest to the optimal solution provided by integer linear programming.

Reynders et al. [21] present a scheme to minimize the packet collision probability, especially in places where devices are distant from the gateway. The authors evaluated their solution in a simulation whose results demonstrate that the packet error rate can be decreased up to 50%. Abdelfadeel et al. [17] proposed an algorithm that optimizes the transmission power and SF in a way that balances the transmission data rate on the network, assuming that all nodes are able to reach the gateway. Different from the aforementioned studies, Jeon and Jeong [25] proposed adaptive changes on both the transmitting power and **up-link** data rate based on the **observed** performance and **enhancements** in the upload channel.

Ramli et al. [8] proposed an adaptive network to switch between LoRaWAN and IEEE 802.11ac [29] protocols in situations of lower or higher data transmission demands, respectively. However, their study did not provide any suggestions regarding the performance enhancement of LoRa’s parameters. The authors highlighted that the Packet Error Rate (PER) for IEEE 802.11ac long-range transmissions are substantially higher than LoRa’s medium and long-range transmissions.

Slabicki et al. [24] propose an adaptive mechanism called FLoRa to configure the main parameters of LoRa networks, focusing on dense IoT scenarios. The authors evaluated the adaptive data rate from LoRa in a synthetic simulation scenario, showing the benefits of using a mechanism that observes and changes the LoRa parameters settings. Other related work evaluated by [24] is Farhad et al. [26], where the authors present two different algorithms called Gaussian-based Adaptive Data Rate (GADR) and Exponential Moving Average-based Adaptive Data Rate (EMA-ADR). The results are promising concerning the packet success ratio, energy consumption, and convergence period compared to [24].

Moysiadis et al. [28] propose a linear regression extension of Adaptive Data Rate (LR-ADR) resource allocation mechanism for the network server side to smooth SNR per gateway and to support LoRa-enabled end-devices to regain the connectivity with the network server faster. They conduct an empirical study to evaluate LR-ADR performance compared to ADR, ADR, EMA-ADR, and G-ADR resource allocation mechanisms. Their results demonstrate that LR-ADR has better performance, an improved Packet Delivery Ratio (PDR), and kept the Energy Consumption per Packet Delivered (ECPD) at lower levels than the aforementioned alternative solutions. The resource allocation mechanism proposed in our work supports the dynamic adjustment of LoRa network performance parameters based on a sliding window to smooth short-term variations and reduce unnecessary network reconfigurations. Our solution focuses on improving SNR, Bit Error Rate, and the number of frequency switches LoRa network performance parameters, while LR-ADR [28] and EMA-ADR solutions focus on improving packet delivery ratio and energy consumption per packet delivered parameters. So, both resource allocation mechanisms can be seen as complementary to each other. To enrich this article, a comparison between our proposal and LR-ADR is present in the evaluation section.

Figueiredo and Franco Silva [14] propose a technique to enhance the efficiency of a LoRa network with practical implications. The authors explore the orthogonal characteristics of different frequency carriers to elaborate an algorithm that periodically measures the SNR signals from the 433 MHz and 915 MHz frequency bands. After evaluating the measurements, a primary node decides between changing the frequency band of all network nodes for the best one or *staying* on the current frequency, according to the SNR values of those channels. However, the obtained results did not show a significant change, as the average improvement on SNR was 4.68% with an elevated *number* of carrier changes. The high frequency of change is a consequence of the decision-making strategy based on the instant measurements of SNR on both bands. During a measuring time, an impulsive noise (i.e., caused by another transceiver) could bring a low distorted SNR value compared to the historical average. In this scenario, the proposed algorithm could make a bad decision due to the impulsive noise on the communication medium. Figueiredo and Franco Silva enhance LoRa networks through a practical approach for signal carrier changes.

Contrasting the methodology presented in our previous work Figueiredo and Franco Silva [14], this article proposes the utilization of an SNR measurements history and a decision maker to decisions based on the average SNR sample instead considering only the last one. Thus, there is a probability of lowering the error rate while deciding due to the filtering effect of the sliding average. Our proposal is simple and presents a linear computational complexity in terms of processing and storage, allowing it to be implemented in resource-constrained IoT nodes. The validation experiments complement the state-of-the-art once a real environment is deployed.

Table 1 summarizes related works based on their optimization goals, and if they (or do not) consider characteristics of energy consumption, application requirements, and targeting LoRa modulation parameters (Spreading Factor—SF, Transmission Power—TP, BandWidth—BW, and Coding Rate—CR). The optimization goals addressed in these studies contribute to improving system performance in terms of increasing of the QoS level by reducing collisions. From the analysis of the state-of-the-art, we identified that there is a lack of studies that exploit the reduction of inter-symbolic interference in communication channels in LoRa networks.

## 3. Proposal

In this section, we introduce the system model and proposal, and highlight the related algorithms and their technological aspects.

### 3.1. Network and System Model

**Figure 1:**
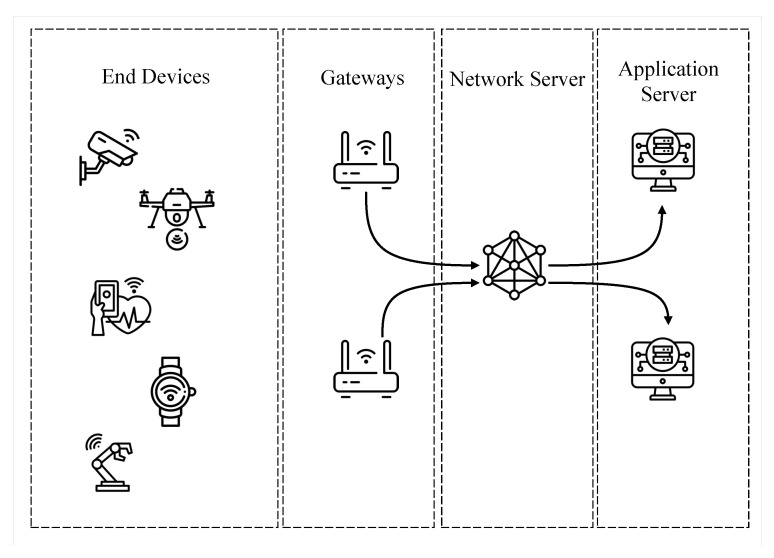
LoRa^TM^ architecture using the LoRaWAN protocol.

The most frequently used LoRa communication architecture is the LoRaWAN (https://lora-alliance.org/about-lorawan/, accessed on 10 December 2022) [13]. LoRaWAN specification considers LoRa radio as the physical layer, it defines the upper layers and the long-range wide area network architecture. Figure 1 depicts the main elements of a LoRaWAN architecture: nodes (or end nodes, or even end devices), gateways (or concentrators), network servers, and application server. Specifically, the application server aims to mediate communication between geographically distant devices. The network server works as a broker for the LoRa application, mediating the communication between the gateways and the application servers. Then, the gateways have the functionality of acting as access points or as device communication coordinators, providing them with connectivity. Finally, there are the end devices (e.g., sensors, smart devices, etc.), which are the nodes that send and receive packets from the Internet (and from the application servers) through the gateways. Versions 1.0.4 and 1.1 of the architecture introduced a Join Server, which is responsible for receiving and processing the join requests that arrive from end devices and authenticating them.

The LoRa^TM^ modulation is related to a proprietary digital spread spectrum designed by *Semtech Corporation*. LoRa is built upon CSS modulation to provide a medium for communication [30]. This scheme takes into account the signal detection sensitivity, which comes with the limitation of the transmission bandwidth. The lower bandwidth has a direct relation with how the modulation works and with the adjustable communication parameters. The designer or engineer of the communication system has to take care of those parameters accordingly with the required network performance [30].

Some advantages that can be observed while using the LoRa modulation are the immunity regarding the multi-path and Doppler effects, the long transmission range, and its low power requirement [30]. Some important configuration parameters are SF, Bandwidth (BW), and Coding Rate (CR). Moreover, it is important to highlight that LoRa-enabled devices are able to execute on ISM (Industrial, Scientific, and Medical) frequency bands of 433 MHz, 868 MHz, and 915 MHz [31].

**Figure 2:**
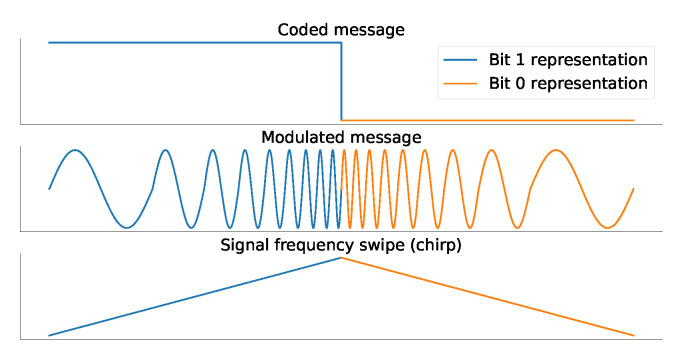
LoRa-based network bit representation.

LoRa has a digital modulation scheme based on the CSS spectral spreading technique to transmit messages, giving high communication rates for range, and reducing power consumption. LoRa modulation is resistant to Doppler and multi-path effects. It consists of 0- and 1-bit representations as linear variations of frequencies. Figure 2 illustrates an example of LoRa 0- and 1-bit representation, the corresponding symbol for each bit, and a representation of the frequency variation of the symbol. The frequency shift rate is controlled by the Spreading Factor (SF) parameter, where higher values mean better signal immunity against noise.

The left side of Equation (1) illustrates the Shannon–Hartley theorem, representing the capacity *C* of a communication link (in bits per second—bps) as a function of *Bandwidth* (*B*) (Hz) (i.e., the amount of data (*packet*) that can be sent and received in an instant *t*), and *Strength* *S* (mW) and *Noise* *N* (mW) ratio (i.e., *S*/*N*) of the signal. The right side of Equation (1) illustrates a simplification of the Shannon–Hartley theorem demonstrating the higher the Bandwidth (*B*), the lower the SNR (Signal to Noise Ratio) of the link (*C*).

(1) $$C=B\times \log_{2}\left(1+\frac{S}{N}\right)\;\to \;\frac{S}{N}\approx \frac{C}{B}$$

The propagation factor in LoRa is built upon base 2 logarithms of the *number of chirps per symbol*, where its settings have a huge influence on the effective network resilience and characteristics. For example, its setting parameters are responsible for coordinating the bit rate of the modulation, the resistance to noise interference, and its decoding. It is possible to highlight the most relevant parameter in LoRa modulation, the bandwidth, or BW. So, a LoRa symbol consists of $2^{SF}$ chirps, responsible for covering the entire frequency band. Considering it, as there are $2^{SF}$ chirps in a symbol, we can conclude that one symbol can effectively encode SF bits of information. The bandwidth BW in LoRa is associated directly with the chirp rate on a network. It is possible to demonstrate it as follows, i.e., $\frac{chirp\;per\;second}{BW\;in\;Hz}$. The chirp has several consequences on modulation behavior, from the signal strength to the adequate bandwidth performed. The symbol rate and bit rate in a given SF are proportional to the bandwidth frequency. Equation (2) correlates the duration of a symbol ($T_{S}$) to *BW* and *SF*.

(2) $$T_{S}=\frac{2^{SF}}{BW}$$

LoRa includes a Forward Error Correction (FEC) to detect and recover from possible bit errors. Thus, *CR* is equal to 4/(4+n), with n∈1,2,3,4, adding redundant bits to perform the error correction/detection. The useful bit rate (*Rb*) is calculated based on BW, SF, and CR link parameters as illustrated in Equation (3). For example, considering a configuration with BW = 125 kHz, SF=7, and CR=4/5 values, the bit rate is equal to Rb = 5.5 kbps. Following Equation (3), Table 2 shows the bit rate for all CR values supported, with the three biggest *BWs* and the available *SFs*.

(3) $$Rb=SF\times \frac{BW}{2^{SF}}\times CR$$

According to Semtech [30], the definition of a LoRa channel rate ($R_{b}$), which is measured in *bits/s*, is represented by the Equation (4). Faber et al. [32] introduced an equation that correlates the Bit Error Rate (BER), on an Additive White Gaussian Noise (AWGN) channel, with the energy per bit-to-noise power spectral density ratio. This can be calculated by $E_{b}/N_{0}$ and is represented by a complementary error function (called function *Q* or function erfc) as shown on Equation (5). Finally, Equation (6) correlates $E_{b}/N_{0}$ with the SNR of a signal.

(4) $$R_{b}=SF\times \frac{1}{\frac{2^{SF}}{BW}}$$

(5) $$BER=Q\left(\frac{\log_{12}\left(SF\right)}{\sqrt{2}}\times \frac{E_{b}}{N_{0}}\right)$$

(6) $$\frac{E_{b}}{N_{0}}\left[dB\right]=SNR\left[dB\right]+10\log_{10}\left(\frac{BW}{R_{b}}\right)$$

Equation (7) is obtained by substituting Equation (4) on Equation (6), which correlates the BER of a LoRa communication on an AWGN channel with the Spreading Factor and the SNR measured from the signal. It is important to highlight that although the equation does not depend on the channel’s signal bandwidth, it depends on the SNR value and the SF.

(7) $$BER=Q\left\{\frac{\log_{12}\left(SF\right)}{\sqrt{2}}\;\cdot \;\left[SNR\left[dB\right]+10\log_{10}\left(\frac{2^{SF}}{SF}\right)\right]\right\}$$

**Figure 3:**
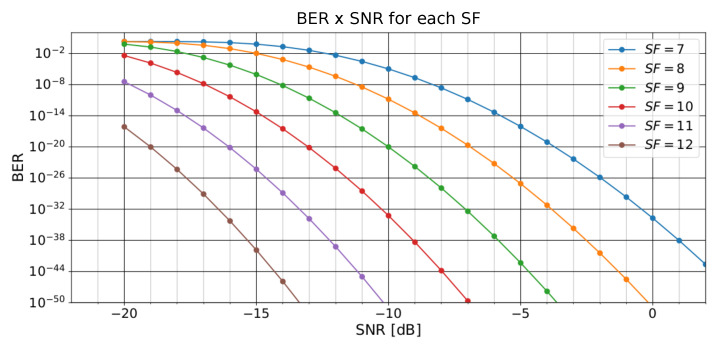
Relation between BER and SNR for a LoRa^TM^ modulation on an AWGN channel considering different SF values.

Finally, Figure 3 illustrates the relationships between BER and SNR for a LoRa modulation on an AWGN channel considering different SF values. We can observe that a higher SNR of a signal provides a lower BER. Additionally, considering the same SNR value, higher SF values performed better on communication.

### 3.2. InstantChange: A Simple Instant Automatic Parameter Changing

The decision to choose the wireless spectrum concerns several metrics. For example, decisions can be taken considering the SNR and received signal power, regarding handover and QoS (Quality of Service) [15]. In IEEE 802.11 and LTE networks, the SNR and RSSI (Received Signal Strength Indication) are the main parameters supported for decision-making concerning proceeding with a handover, the bandwidth selected, and the channel configuration to be used [33].

In the context of long-range networks, there exist several sources of signal that can generate interference, deteriorate quality, and harm communication between two points. In this work, we investigate the signals collected in a real LoRa network. We used the collected data to improve an existing cognitive protocol to support the identification of the best configuration and frequency range in a given moment (scenario). Since SNR is a good physical-layer metric to evaluate the quality of the received signal, we considered the SNR metric in our proposal to make configuration changes in LoRa nodes. To achieve this goal, we present our implementation of Cognitive LoRa named InstantChange (see Algorithm 1).
**Algorithm 1:** Cognitive LoRa for two (or more) frequencies. Proposed by [14]./*(Range of frequencies evaluated. ( */**1** F={433,915}//(Range of spread spectrum evaluated. (**2** SF={10,11,12}//(Range of bandwidth evaluated. 125/250 kHZ for EU433 or 125/500 kHz for AU915 [31].(
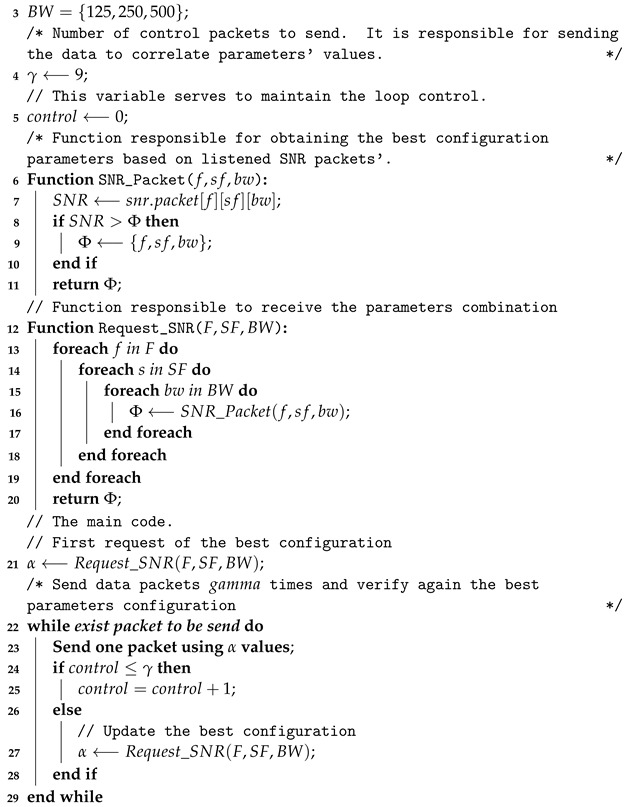


Algorithm 1 shows the behavior of the InstantChange core. This algorithm is responsible for performing the configuration based on network-awareness, changing the parameters of frequency, spread spectrum, and bandwidth. It is important to highlight that this algorithm considers only the current instant to perform (or not to perform) a change of configurations in the nodes. The main objective of this proposal is to maximize the SNR. Considering the intelligence behind the algorithm’s logic, the variables and steps can be presented as follows: a node is responsible for sending LoRa data packets/datagrams γ times using the frequency, spread spectrum, and bandwidth (F,SF,BW) settings determined as the best configuration, represented for α.

Once the number of data packets to be sent is reached, a new evaluation is carried out. To keep the parameters’ values synchronized by all nodes, a control packet is generated to inform that it is necessary to change to another configuration. So, the control packet about the request is generated for each configuration of F,SF,BW, using the function Request_SNR. This function is responsible to send a control packet and measure the SNR through the function SNR_Packet. Once the values are stored, the SNR_Packet provides the inputs about the best configuration and returns it to the main code, where the algorithm decides whether or not to make a shift on the α parameters. The γ and control variables were used for controlling the experiments. The control variable begins with 0, and it is incremented by 1 unit each round of sending a packet until its value is less or equal to γ values, i.e., 9. This loop control allows us to send 10 packets before changing the configuration parameters. The φ variable stores the best configuration parameters (frequency, spreading factor, and bandwidth) based on the listened SNR packets’. The possible φ configurations are described in Table 2.

### 3.3. SlidingChange: A Proposal of a Sliding Window for Automatic Parameter Changing

The proposed *SlidingChange* algorithm comprises two different stages: (i) the data transmission step and (ii) the network control step. In the first stage, a sequence of messages is sent by the secondary nodes trying to reach the primary node. The only goal of doing that is to record SNR values in the network during an instant *t* (represented by a call to **sendDataPacket() function** in the Algorithm 2). After reaching a predefined amount of messages, the system starts the control stage. While in this step, the primary node sends some messages containing the next configuration to be tested and then waits for an answer from the secondary nodes. The configuration parameters consist of SF, BW and $F_{carrier}$ (carrier frequency). The answer must come with the previous configuration and, after a previously set time frame (set by the primary node), every device changes its configuration. This process repeats itself until all the possible configurations are fully tested. With these data, the primary node computes an SNR sliding window value using the secondary nodes’ responses compared with other configurations to decide on changing or not the carrier frequency parameter. Even if the algorithm changes the parameters, the scanning process keeps testing all possible configurations because, in the future, the discarded parameters could become the optimal ones again, and it would be possible to reanalyze these data.

**Figure 4:**
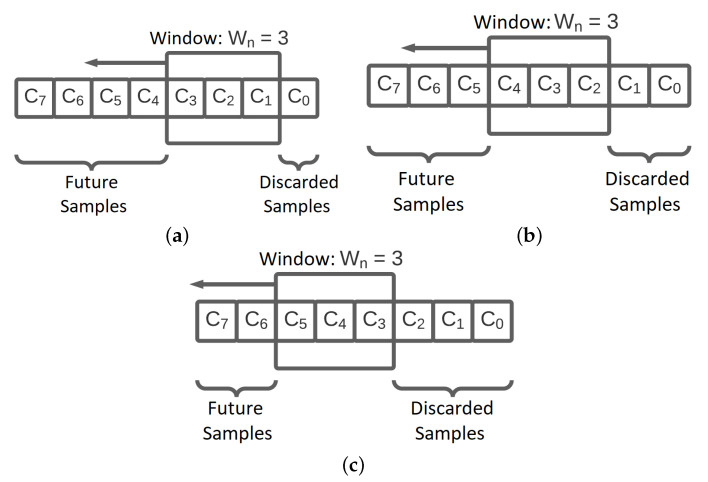
Sliding window operating principle within the scope of this proposal. (**a**) Stage 1: First window state in a given time. (**b**) Stage 2: Window state when an element was discarded. Window elements were relocated, and another was added at the beginning of the window. (**c**) Stage 3: Window state with element drop, displacement, and sample inclusion occurring again.

This study observed the behavior of the sliding window algorithm, which considered the SNR values obtained from the data packets. The configurations considered were those with windows of length equal to W=10,20,30, and 40 samples because it was desired to compare the different window lengths. Figure 4 presents the operating principle of the sliding window, which can also be seen on Algorithm 3.

Algorithm 3 presents the sliding operation of the values’ window. For a sliding window of $W_{n}$ samples, the samples are moved one unit each time. For example, considering one window of three samples, e.g., $W\left[3\right]\leftarrow \left[C_{2},C_{1},C_{0}\right]$, the slide operation would be $W\left[3\right]\leftarrow \left[C_{2},C_{2},C_{1}\right]$. Then, a new message packet is read, and its SNR value is written at the beginning of the window, so in our example, it would be $W\left[3\right]\leftarrow \left[C_{3},C_{2},C_{1}\right]$. Note that once the sliding operation is finished, the sample $C_{0}$ is then discarded.
**Algorithm 2:** Moving average in cognitive LoRa.
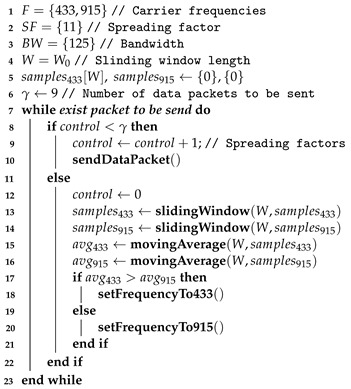


The process described above is detailed in Figure 4. Figure 4a presents the first step of the sliding window parameter change solution. The samples $C_{1}$, $C_{2}$, and $C_{3}$ are added to the sliding window, the sample $C_{0}$, which is part of the window, is discarded, and other samples will be collected (future samples). In practice, they were not recorded on the system memory yet. In the second step, illustrated in Figure 4b, a newer sample $C_{4}$ arrives at the micro-controller and the window is moved to the left, writing this sample in the data set and discarding the sample $C_{1}$. Then, this process repeats in the third step as shown in Figure 4c.
**Algorithm 3:** Sliding window of length equals to $W_{n}$.
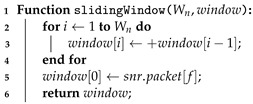


Algorithm 4 describes the logic behind the computation of the average values. An accumulator is created and then values from all sliding window samples are added to it. This sum is then divided by the number of values added, and then the algorithm returns the result. Since the Algorithm 4 only computes the average of a set of values, the combined use of Algorithms 3 and 4 provide the computation of a moving average. Hence, Algorithm 2 uses those two algorithms and runs the tests to provide a decision regarding the change or not of the carrier frequency, according to the environmental conditions.
**Algorithm 4:** Moving average values for windows’ length equals to $W_{n}$.
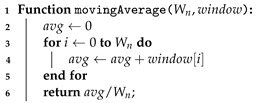


## 4. Evaluation

**Figure 5:**
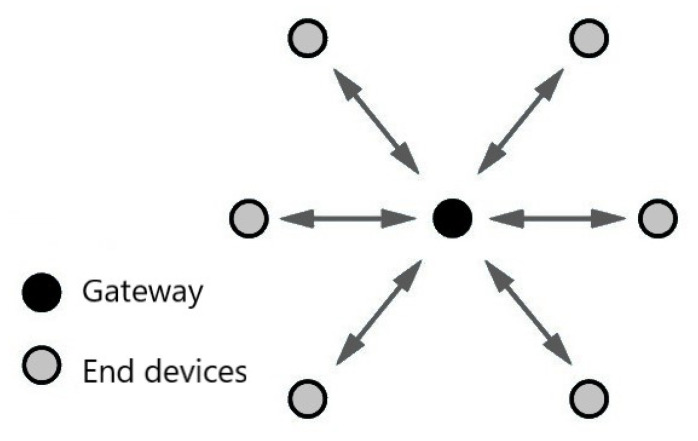
An example of LoRa’s network topology setup used in the tests.

**Figure 6:**
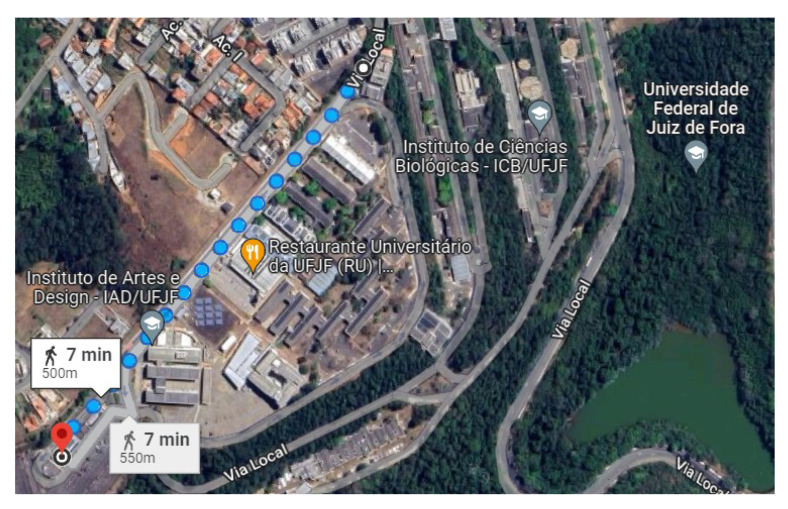
Testbed scenario. From Computer Science Department to CRITT (Technological and Innovation Center).

During the development of this work, we deployed a small LoRa network. The network illustrated in Figure 5 comprises one primary node together with other nodes, and they form a graph with a star topology. We developed two algorithms for operating the primary (gateway) and secondary devices. Figure 6 shows the scenario under our university campus where the test was deployed. The distance between the nodes was nearly 500–700 m.

### 4.1. Prototype

**Figure 7:**
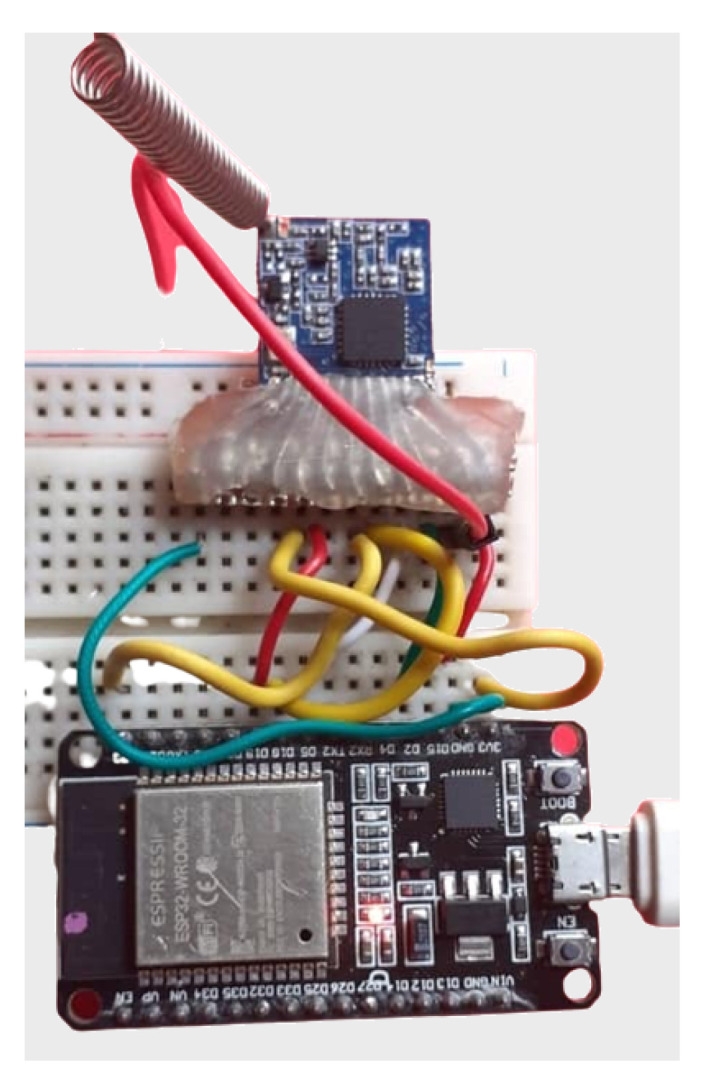
LoRa sensor/client and gateway nodes.

**Figure 8:**
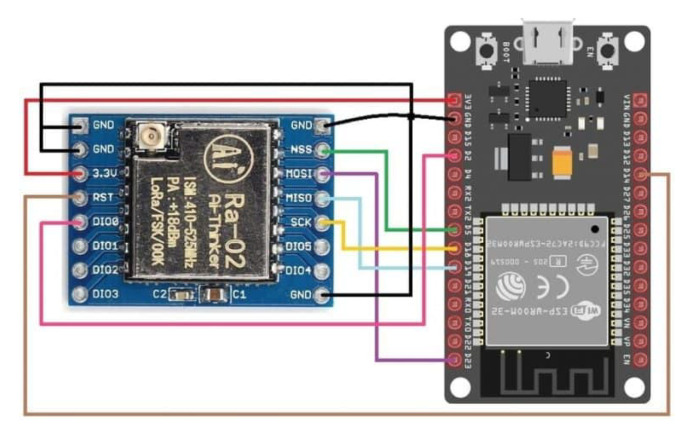
LoRa sensor/client and gateway nodes GPIO connections.

As mentioned, a testbed was created to conduct the evaluation. It is composed of LoRa nodes client/gateway prototypes and a mobile application to configure the nodes, responsible for running the experiments and collecting the measures. Figure 7 and Figure 8 show the client and gateway node connections with details.

### 4.2. Mobile App for Command and Control

**Figure 9:**
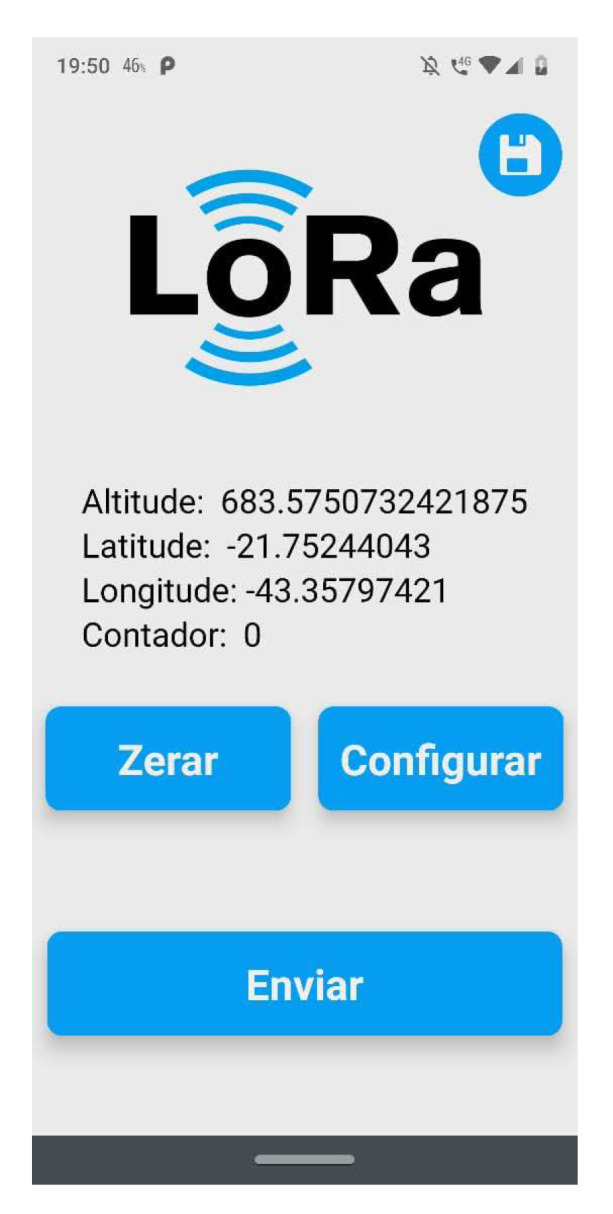
Remote control screen on mobile app.

**Figure 10:**
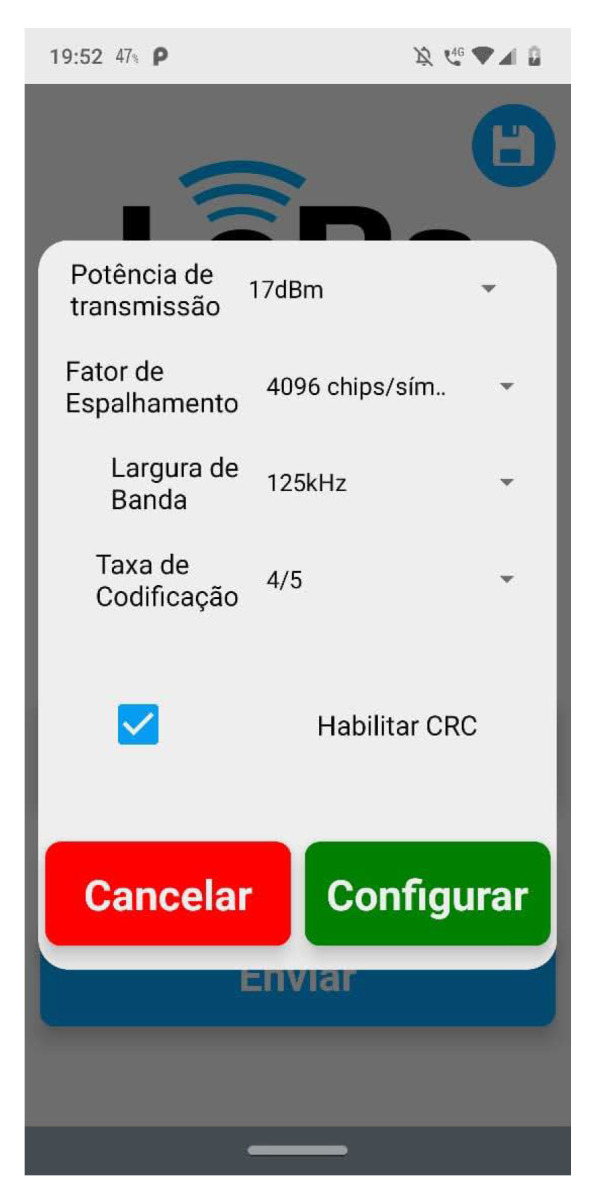
Settings screen on mobile app.

To collect data from our experiments, a mobile app was built. This solution was responsible for connecting to LoRa nodes and sending and receiving data about settings and measures. Figure 9 and Figure 10 show the main screen about sending configuration settings to the nodes. To connect to the nodes, a Wi-Fi interface from ESP32 was used.

### 4.3. Results

**Figure 11:**
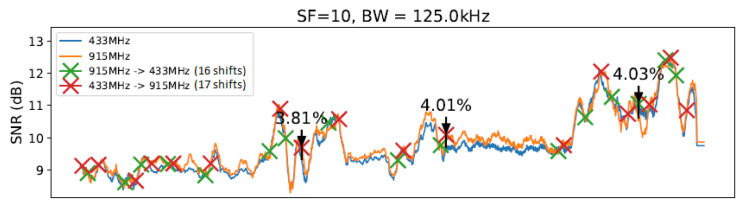
Without SlidingChange (InstantChange) W=0.

**Figure 12:**
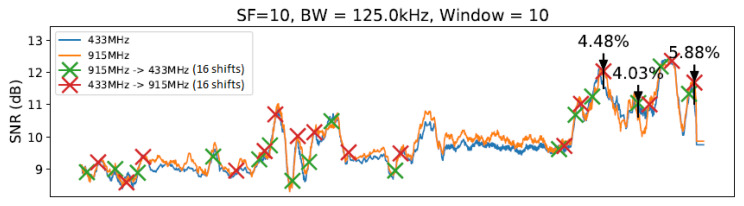
SlidingChange with W=10 elements.

**Figure 13:**
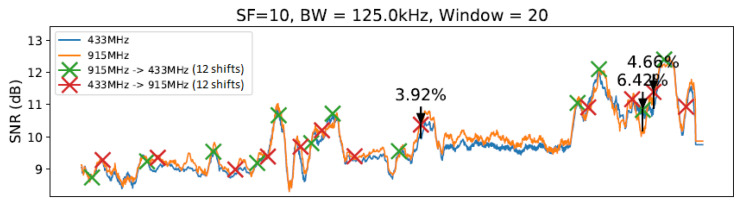
SlidingChange with W=20 elements.

**Figure 14:**
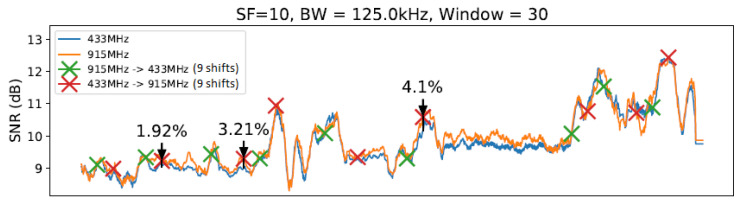
SlidingChange with W=30 elements.

To conduct our experiment, we created a realistic scenario comprising an ESP32 client and a gateway equipped with SEMTECH SX1276 and SX1278 modules implementing a LoRa physical layer. We established communication between the client and gateway nodes under 433 MHz and 915 MHz frequencies (as described in [14]) using two different antennas. We considered 10, 11, and 12 (we ignored 7, 8, and 9 SF values due to previous measurements and the correlation between lower SF values had implied in worse transmitter sensitivities [30]) SF values in this evaluation. Concerning bandwidth (BW), we considered the following values defined in LoRaWAN specification. We decided to use a fixed value of 4/5 ratio for the coding rate (CR) throughout all experiments in order to simplify the execution of the tests. Figure 6 illustrates the evaluation scenario involving communication from the Computer Science Department to the CRITT Innovation Center on the Federal University of Juiz de Fora campus. The obtained results are summarized in Table 3. Since there are correlations between SNR values for distances from 1 and 800 m, we can conclude that in the evaluated outdoor environment, measures of approximately 10 m of distance between the nodes are enough to demonstrate the need for node-aware parameter changes to improve SNR. The graphs from Figure 11, Figure 12, Figure 13 and Figure 14 illustrate the change/shift points, and the biggest three SNR improvements were highlighted with an arrow in each figure (We only present a sample of the measures at this moment. We complement these results later using tables).

The graphs introduced by this section show a comparison of both techniques: without any windows, referring to the technique by [14] (Figure 11), and with windows of 10 and 20 units of length (Figure 12 and Figure 13), using SF=10 and BW=125 kHz.

As illustrated in Figure 11, Figure 12, Figure 13 and Figure 14, we noted that for the presented configuration, there is a direct correlation between the maximum SNR values and the window size. As the length of the window becomes larger, there are slightly higher maximum SNR values measured, especially considering the 20 elements’ window.

Considering the previous related work, InstantChange and SlidingChange algorithms were compared with LR-ADR to complement and enrich our analysis. All the algorithms were implemented to conduct the comparison. Despite LR-ADR being developed to select the best network server in a LoRa network with multiple gateways, we compare our approach with it, considering the gain in terms of SNR. The LR-ADR uses simple linear regression to smooth the SNR signal from the last packets received to introduce the LR-ADR. The authors consider, by default, 10 SNR values for each gateway per end device. In our case study, in a real testbed with one gateway and real data collected, we consider different SF, BW, and Frequency values as a base to apply the LR-ADR and configure it. The configuration shifts and SNR metrics were computed and compared with our proposal.

Considering LR-ADR algorithm, the SNR is calculated in time *T* as follows: $SNR_{T}=\beta T+\alpha$ where $SNR_{T}$ is the SNR at time *T*, and α is equal to $\alpha =\bar{SNR}-\left(\beta \bar{T}\right)$. The β is represented by:

(8) $$\beta =\frac{\sum_{i=1}^{n}\left(T_{i}-\bar{T}\right)\left(SNR_{i}-\bar{SNR}\right)}{\sum_{i=1}^{n}{\left(T_{i}-\bar{T}\right)}^{2}}$$

To estimate the next expected SNR values and choose the parameters’ values, the authors use

(9) $$SNR_{N}=\beta \left(T+T_{period}\right)+\alpha$$

More details about the LR-ADR algorithms can be found in [28].

Table 3 shows the best result for each parameter value combination in the experiment at a specific moment, and Table 4 shows the number of shifts of each setting to another. Table 3 shows all observed configurations and their corresponding SNR gains. The average from all 27 measurements taken on the given setup (i.e., windowless, a window of 10 units of length, etc.) is displayed in the “Average” column. Measurements 1, 2, and 3 show the first, second, and third biggest SNR gain variations from each setup with a sliding window, respectively. The highlighted values show the highest SNR gain (in this case, 11.89%) and the best gain average (of 4.60%) observed.

From Table 3, it is possible to note a slight rise in the SNR gain for the technique with a window using W=10 and a smaller rise for the window with W=20. The window with W=30 presented a significantly smaller gain compared with the previous two window lengths. Then, the window with W=40 scored the smallest gain of all techniques. Observing the data on Table 3, it is possible to note that the window with W=20 scored the best results from the entire table, reaching an SNR gain of 11.89%. However, as the average values show, the window with W=10 achieved the best gain consistency. Finally, we can highlight that LR-ADR obtained a result close to not using windows. Considering that the LR-ADR is based on simple linear regression with smoothing for the last SNR measure, the results are very intuitive, keeping the tendency of the next SNR value, which is not always the best configuration.

Table 4 shows the frequency of the shifts that occurred and the average of nine measurements from the changes for each setup. The highlighted values represent the biggest and smallest frequency shift averages. From an analysis of Table 4, it is possible to note that the SlidingChange technique with the window length equal to 10 elements, considering the average values, reached the highest number of carrier changes, scoring even higher values than the InstantChange technique. The technique with W=20 elements had a moderate reduction, while the windows with 30 and 40 elements presented the most significant changes compared to the InstantChange technique. It is worth noting that the technique with 40 elements scored a reduction ratio of more than 50% on carrier changes compared with the InstantChange technique. The LR-ADR obtained the worst result, generating the highest number of configuration shifts, even more than the configuration with window W=10. We believe that this is due to the use of the trend of the value of the next SNR found by the linear regression method simple, which is not always correct in environments with mobility, as is the case of the evaluated environment.

Based on the data from Table 3 and Table 4, Table 5 illustrates the increase in SNR gains by percentage when compared with the results obtained from the InstantChange technique presented in [14]. For each window length evaluated in this paper, it showed a reduction in the number of changes in the carrier modulation compared with the InstantChange technique. From the analysis of Table 5, we observed that the window with W=10 is the one that achieved the best SNR gains, scoring an improvement of 44.89% compared with the InstantChange technique. However, it reached a higher number of frequency changes, as 37.80% more changes were recorded regarding that parameter.

In contrast, compared to the InstantChange technique, the sliding window of W=40 is the one that achieved the highest reduction on the average number of carrier changes, scoring 53.25% fewer changes. However, the sliding change technique showed the smallest SNR gain, scoring a reduction of 8.92% of the average SNR gain.

The window with W=0 was represented by the InstantChange technique. From the analysis of the results, we concluded that the larger sliding window does not always mean a higher SNR gain. We identified the most efficient setups have between 0 and 30 units of length. The sliding window of 20 units of length could be considered the one with the best efficiency ratio since it achieved a considerable reduction in the carrier frequency changes and still had an SNR gain. Considering the LR-ADR, we would like to highlight that the proposed technique by [28] was created to be applied in a LoRa network with more than one gateway. Once it is not our scenario, the LR-ADR was evaluated, keeping the parameters (i.e., SF, BW, Frequency) values or shifting to another one. This particularity of our evaluation and proposal objectives could have influenced the LR-ADR performance’s final result.

## 5. Conclusions

In this paper, we described the results from [14] using the InstantChange technique. We also presented a novel technique called SlidingChange, which obtained improvements on Bit Error Rate and Signal-to-Noise Ratio values. Those improvements can be demonstrated considering the relationships shown in Figure 3. Moreover, our work proposed techniques that considerably reduced the **configuration** (parameter) changes, improving the LoRa’s network quality.

The sliding window technique, SlidingChange, achieved significant improvements compared to the windowless technique, InstantChange, and it performed better than the static LoRa configuration, which does not change any parameter over time. We noted that due to the filtering properties of the sliding window, the number of changes becomes lower when the window length is increased. However, the same effect could make the algorithm perform bad frequency changes, as illustrated by the results from the smaller sliding windows.

From the analysis of the average of SNR gain and the number of changes shown in Table 3 and Table 4, we can conclude that the best results (when considering these results only) were achieved by the sliding window of W=10 for the SNR gain and the sliding window of W=40 for the number of changes.

The results related to the window technique presented in Table 5 show that the window length W=20 achieved the best balance between SNR gains and reduction of the number of carrier changes. This configuration achieved, when compared with the windowless technique, an improvement of 36.73% on the SNR gain and 16.26% fewer frequency changes.

The hypothesis that the sliding window always reduces the number of frequency changes was tested as true only for the windows of 20, 30, and 40 units of length, as the window of 10 units scored a higher number of changes. Nevertheless, the hypothesis that a sliding window for the moving average of SNR as an input to the decision-making regarding LoRa’s carrier frequency changes was partially confirmed, because longer window lengths reduced the gain instead of increasing it. Therefore, the proposed technique demonstrated that for a desired window length range there was an increase compared with the windowless technique. Those improvements represent advancements in LoRa’s communication quality, as highlighted in Figure 3 that shows the relation between a decreasing BER and an increasing signal’s SNR value. Moreover, the sliding window technique was better than the windowless technique, noting that those better results, depending on the window length and parameter, are being taken into consideration (number of changes or SNR gain), whichever is more important for some appliances or others.

Finally, according to [34], until 2020 it was expected for there to be more than 25 billion Internet-connected devices. Only in the United States, as pointed out by [35], it is believed that the IoT market spending was around $245 billion and would reach $8.131 trillion in 2030. Studies such as this one, which aim to enhance devices’ efficiency and effectiveness in connecting and interacting with computer networks, are relevant to support improving the process of devices’ interconnections.

In future work, we intend to investigate other methodologies to support the decision-making process of the frequency changes. For example, using other types of sliding windows, calculating the impact of those setups and the length of the windows, as well as calculations through an exponential moving average. The authors also intend to investigate the impact of the proposed techniques, comparing them with other technologies and frequency spectra beyond LoRa, such as LPWAN. Future works can consider evaluating the pros and cons of using sliding window techniques compared to other optimization techniques based on ADR. We also intend to conduct an experimental study to analyze the trade-offs between physical-layer and network performance parameters such as energy efficiency and latency.

## Figures and Tables

**Table 1:** Summary of related work and its mechanisms.

LoRa Improvement Proposal	Year	Optimization Goal	Cognitive	LoRa Modulation Parameters	
			**Real-Time Settings**	**SF**	**BW**
Bor et al. [19]	2016	Maximize the transmission range and energy-saving		X	X
Bianchi et al. [5]	2018	Address the unfair LoRaWAN characteristic		X	
Valach and Macko [22]	2019	Reduce collisions			
Lima et al. [11]	2017	Reduce collisions			
Moraes et al. [23]	2019	Maximize QoS		X	
Reynders et al. [21]	2018	PDR and throughput		X	
Abdelfadeel et al. [17]	2018	Improve LoRaWAN capacity and reduce collisions		X	X
Jeon and Jeong [25]	2020	Maximize the channel utilization and reduce collisions		X	
Ramli et al. [8]	2020	Improve the noise resilience and PDR		X	
Slabicki et al. [24]	2018	SNR smoothing		X	
Moysiadis et al. [28]	2021	Improve packet-delivered		X	
Farhad et al. [26]	2020	Improve scalability, maximize SNR		X	
Figueiredo and Franco Silva [14]	2020	Improve scalability, maximize SNR and BER	X	X	X
**Proposed mechanism**	**2023**	**Improve scalability, maximize SNR and BER**	X	X	X

**Table 2:** Relationships between Spreading Factor (SF), Bandwidth (BW), and maximum throughput in kbps.

		Bandwidth (kHz)												
		125				250				500				
		CR												
		4/5	4/6	4/7	4/8	4/5	4/6	4/7	4/8	4/5	4/6	4/7	4/8	
**SF**	**7**	5.5	4.6	3.9	3.4	10.9	9.1	7.8	6.8	21.9	18.2	15.6	13.7	***kbps***
	**8**	3.1	2.6	2.2	2.0	6.3	5.2	4.5	3.9	12.5	10.4	8.9	7.8	
	**9**	1.8	1.5	1.3	1.1	3.5	2.9	2.5	2.2	7.0	5.9	5.0	4.4	
	**10**	1.0	0.8	0.7	0.6	2.0	1.6	1.4	1.2	3.9	3.3	2.8	2.4	
	**11**	0.5	0.4	0.4	0.3	1.1	0.9	0.8	0.7	2.1	1.8	1.5	1.3	
	**12**	0.3	0.2	0.2	0.2	0.6	0.5	0.4	0.4	1.2	1.0	0.8	0.7	

**Table 3:** Summary of the three biggest SNR gains for each setup and their respectively averages.

Window	Experiment	BW									Average
		125 kHz			250 kHz			500 kHz			
		SF			SF			SF			
		10	11	12	10	11	12	10	11	12	
LR-ADR [28]	1	4.01%	3.22%	4.14%	3.10%	4.37%	2.92%	2.98%	2.21%	3.78%	3.05%
	2	3.92%	2.75%	3.33%	3.34%	2.54%	2.88%	2.34%	2.02%	2.09%	
	3	4.03%	4.04%	3.17%	3.16%	2.43%	2.88%	2.03%	2.45%	2.13%	
Windowless	1	4.03%	2.91%	4.74%	4.54%	4.77%	3.72%	2.98%	2.31%	3.88%	3.17%
	2	4.01%	2.50%	3.60%	3.80%	2.56%	2.98%	2.44%	2.19%	2.49%	
	3	3.81%	2.45%	3.53%	3.76%	2.23%	2.92%	2.03%	2.10%	2.37%	
w = 10	1	5.88%	9.79%	7.85%	5.90%	3.33%	5.29%	9.33%	3.31%	5.17%	**4.60%**
	2	4.48%	5.11%	4.49%	3.73%	2.65%	4.91%	4.50%	2.81%	3.98%	
	3	4.03%	4.64%	3.90%	3.36%	1.97%	4.90%	2.59%	2.40%	3.80%	
w = 20	1	6.42%	4.09%	**11.89%**	3.00%	3.32%	5.04%	8.14%	3.33%	8.20%	4.34%
	2	4.66%	4.04%	5.50%	2.50%	2.89%	4.81%	2.54%	2.67%	5.06%	
	3	3.92%	3.09%	4.07%	2.02%	2.68%	4.35%	2.30%	2.22%	4.36%	
w = 30	1	4.10%	4.32%	5.09%	5.90%	4.74%	4.83%	4.26%	5.39%	4.36%	3.61%
	2	3.21%	3.70%	4.67%	2.84%	1.47%	4.51%	2.99%	5.33%	4.17%	
	3	1.92%	2.48%	3.83%	1.96%	1.44%	3.64%	2.30%	2.80%	1.19%	
w = 40	1	3.21%	5.91%	7.39%	4.50%	2.56%	3.53%	2.97%	4.49%	2.60%	2.89%
	2	2.06%	3.12%	4.01%	2.17%	1.46%	3.21%	2.96%	2.31%	2.29%	
	3	1.29%	2.58%	1.72%	1.31%	1.39%	3.02%	1.45%	2.26%	2.24%	

**Table 4:** Summary of the number of configuration changes for each setup.

Window/Technique	BW									Average
	125 kHz			250 kHz			500 kHz			
	SF			SF			SF			
	10	11	12	10	11	12	10	11	12	
LR-ADR [28]	46	52	55	58	57	42	43	39	41	48.111
Windowless	33	32	32	38	37	24	15	13	22	27.333
w = 10	32	52	38	46	46	39	30	28	28	**37.667**
w = 20	24	29	22	23	32	22	15	21	18	22.889
w = 30	19	19	17	19	18	17	12	13	15	16.556
w = 40	14	17	11	13	14	15	9	9	13	**12.778**

**Table 5:** Results of SlindingChange technique compared with average results from the InstantChange technique.

Window	SNR Gain	Number of Changes
10	44.89%	37.80%
20	36.73%	−16.26%
30	13.77%	−39.43%
40	−8.92%	−53.25%
